# Immune Functional Analysis of *Chitin Deacetylase 3* from the Asian Citrus Psyllid *Diaphorina citri*

**DOI:** 10.3390/ijms21010064

**Published:** 2019-12-20

**Authors:** Hai-Zhong Yu, Ning-Yan Li, Bing Li, Shahzad Toufeeq, Yan-Xin Xie, Yu-Ling Huang, Yi-Ming Du, Xiang-Dong Zeng, Bo Zhu, Zhan-Jun Lu

**Affiliations:** 1 School of Life Sciences, Gannan Normal University, Ganzhou 341000, China; yuhaizhong1988@163.com (H.-Z.Y.); 18435203582@163.com (N.-Y.L.); 18970750806@163.com (Y.-X.X.); huangyuling2333@163.com (Y.-L.H.); dym1009@163.com (Y.-M.D.); zxdong224@163.com (X.-D.Z.); nczb615@163.com (B.Z.); 2 National Navel Orange Engineering and Technology Research Center, Gannan Normal University, Ganzhou 341000, China; 3 Institute of Sericulture, Anhui Academy of Agricultural Sciences, Hefei 230031, China; libing2504@163.com; 4 School of Life Science, Anhui Agricultural University, Hefei 230031, China; toufeeq@163.com

**Keywords:** *Diaphorina citri*, *chitin deacetylase 3*, expression profiles, immune response

## Abstract

Chitin deacetylase (CDA) is a chitin degradation enzyme that strictly catalyzes the deacetylation of chitin to form chitosan, which plays an important role in regulating growth and development, as well as the immune response. In this study, a *chitin deacetylase 3* gene (*CDA3*) was identified with a complete open reading frame (ORF) of 1362 bp from the genome database of *Diaphorina citri*, encoding a protein of 453 amino acids. Spatiotemporal expression analysis suggested that *D. citri* *CDA3* (*DcCDA3*) had the highest expression level in the integument and third-instar nymph stage. Furthermore, *DcCDA3* expression level can be induced by 20-hydroxyecdysone (20E). Injection of *Escherichia coli* and *Staphylococcus aureus* induced the upregulation of *DcCDA3* in the midgut, while DcCDA3 was downregulated in the fat body. After silencing *DcCDA3* by RNA interference, there was no influence on the *D. citri* phenotype. In addition, bactericidal tests showed that recombinant DcCDA3 inhibited gram-positive bacteria, including *S. aureus* and *Bacillus subtilis* (*B. subtilis*). In conclusion, our results suggest that *DcCDA3* might play an important role in the immune response of *D. citri*.

## 1. Introduction

The Asian citrus psyllid (ACP), *Diaphorina citri* Kuwayama, is a prominent pest of citrus crops worldwide. In addition, *D. citri* is a vector of bacteria in the genus *Candidatus* Liberibacter, the presumable causative agents of huanglongbing (HLB) disease in citrus [1]. To date, effective control of *D. citri* has become a critical strategy to prevent the spread of HLB [2]. In recent years, the control of *D. citri* has been mainly dependent on chemical insecticides. However, their improper use has led to the poisoning of farmers, degradation of land and water, and increased levels of dangerous chemicals in the food supply [3,4]. Therefore, alternative strategies for *D. citri* control should be explored to reduce the dependence on chemical insecticides. In previous research, many potential genes have been identified and applied to pest control combined with RNA interference (RNAi). Zhao et al. found that the *chitin synthase 1* (*CHS1*) gene can be used as a specific target to control agricultural insects by plant-mediated RNAi [5]. In *Toxoptera citricida*, silencing of *TcCHS* through plant-mediated dsRNA feeding did not allow most dsRNA-fed nymphs to molt to the next stage [6]. Similarly, the knockdown of key genes was applied to the *D. citri* control. Yu et al. revealed that knockdown of the endogenous muscle protein 20 gene will lead to high mortality [7]. Killiny et al. also reported that knockdown of four cytochrome P450 family genes will reduce oxidase activity and insecticide resistance in *D. citri* [8]. These results indicated that effective gene targeting might be useful for the control of *D. citri*.

Chitin, a polymer of *N*-acetylglucosamine, is mainly distributed in the cuticle exoskeleton, tracheal cuticle, and peritrophic matrix in insects [9]. Its biosynthesis and degradation are important for insect growth and development, especially for procuticle integrity, assembly and stabilization of the chitin-less epicuticle. Chitin has two different crystalline forms, α-chitin and β-chitin, while a third form, γ-chitin is a combination of both α-chitin and β-chitin [10]. Antiparallel alignment may contribute significantly to the mechanical strength and stability of arthropod cuticles that predominantly contain α-chitin. In contrast, there is an increase in the number of hydrogen bonds formed with water molecules in β- and γ-chitin [11]. Although chitin is critical for insects’ growth and development, humans and higher animals do not possess a chitin metabolism system. Therefore, many key genes in insect chitin metabolism have been researched as important insecticidal targets. 

Chitin deacetylases (CDAs, EC 3.5.1.41) are part of the carbohydrate esterase family 4, which can catalyze the removal of acetyl groups from the chitin polymer to form chitosan [12]. The first insect CDA was cloned from the midgut cDNA library of *Trichoplusia ni*, and it was discovered that this enzyme was related to the formation of a midgut peritrophic membrane with strong chitin-binding activity [12]. Based on the presence and order of the domains and the overall degree of similarity, insect CDAs can be divided into five groups (Groups I–V). Group I and Group ІІ CDAs encode a chitin-binding peritrophin-A domain (CBD) followed by a low-density lipoprotein receptor class A (LDLa) domain and the CDA domain. Group ІІІ and Group ІV CDAs contain CBD and CDA domains. However, Group V CDAs seem to have retained only the CDA domain [13]. In recent years, CDAs have been identified from various insects, including *Drosophila melanogaster*, *Locusta migratoria*, *Cnaphalocrocis medinalis*, *Nilaparvata lugens*, and *Tribolium castaneum* [14,15,16,17,18]. Insect CDAs show different expression patterns according to their developmental stages and tissues. Xi et al. revealed that *N. lugens CDA3* has a high expression in the midgut, while *CDA1*, *CDA2*, and *CDA4* show the highest expression in the epidermis; injection of CDA1, CDA2, and CDA4 dsRNA all led to molting failure and high mortality [17]. In *T. castaneum*, knockdown of *TcCDA1* and *TcCDA2* led to developmental disturbances and abnormal phenotypes, while no visible phenotypic changes were observed after injection of dsRNAs for *TcCDA3*–*9* [13]. However, the functions of CDAs have not been identified in *D. citri*. 

In this study, we identified a cDNA encoding the whole ORF of *CDA3* from the genomic database of *D. citri*. The *DcCDA3* sequence was analyzed by bioinformatics, and its molecular characteristics and functions were predicted. The expression profiles of *DcCDA3* in different developmental stages and tissues, as well as the response to 20E treatment, were determined. In addition, we analyzed the expression levels following immune challenges with *S. aureus* and *E. coli*, and the biological functions of the recombinant DcCDA3 protein. These results will lay a foundation for further research on the functions of *DcCDA3*.

## 2. Results

### 2.1. Bioinformatics Analysis of the DcCDA3 Sequence

**Figure 1:**
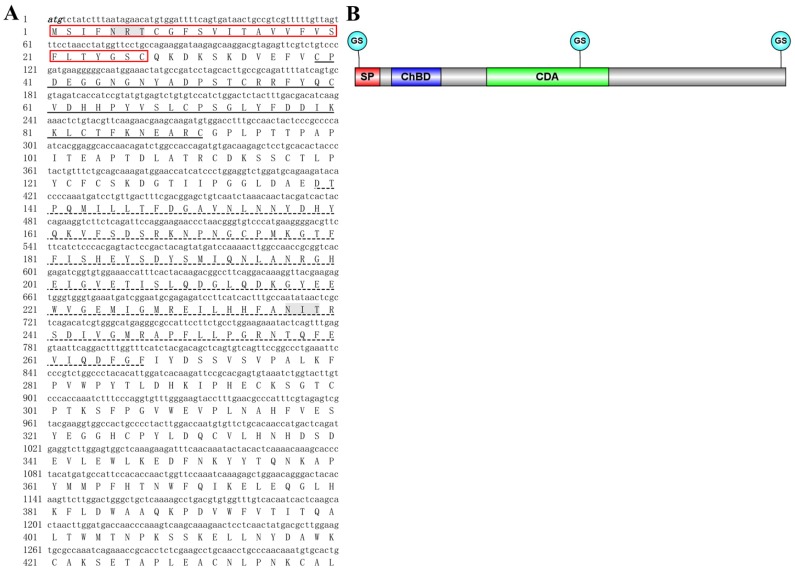
Bioinformatics analysis of the *D. citri chitin deacetylase 3* (*DcCDA3*) cDNA sequence. (**A**) Complete nucleotide and deduced amino acid sequence of the *DcCDA3* cDNA. Numbers on the left side represent nucleotide and amino acid positions. The initiation codon (ATG) and termination codon (TAA) are indicated in black italics. The signal peptide is represented in the red box. The chitin-binding domain (ChBD) and polysaccharide deacetylase-like catalytic domain (CDA) are represented by a black thick line and a black dotted line, respectively. The three potential *N*-glycosylation sites are shaded. (**B**) Structural domain of DcCDA3 was predicted using the IBS 1.0 software. The red box indicates the signal peptide, the blue box indicates the chitin-binding domain, and the green box indicates the polysaccharide deacetylase-like catalytic domain. The grey region represents amino acid sequences. The three blue circles represent *N*-glycosylation sites.

**Figure 2:**
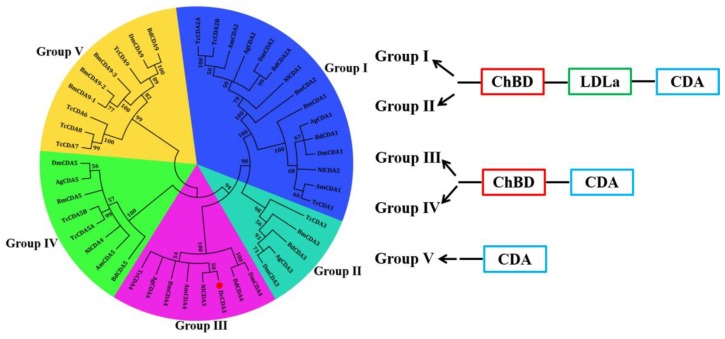
Phylogenetic relationships of DcCDA3 in different insect species using the neighbor-joining method with a bootstrap value of 1000. Chitin deacetylases were from *Tribolium castaneum* (Tc), *Apis mellifera* (Am), *Anopheles gambiae* (Ag), *Drosophila melanogaster* (Dm), *Bactrocera dorsalis* (Bd), *Nilaparvata lugens* (Nl), *Bombyx mori* (Bm), and *Diaphorina citri* (Dc). Group I and Group II CDA contain a chitin-binding domain (ChBD), a low-density liporprotein receptor class A domain (LDLa), and a polysaccharide deacetylase-like catalytic domain (CDA). Group III and Group IV CDA contain ChBD and CDA domains. Group V CDA contains only the CDA domain. The red dot indicates *D. citri* CDA3.

The cDNA sequence of *DcCDA3* (GenBank accession number: XM_008481889.1) contains an ORF of 1362 bp encoding a protein of 453 amino acid residues with a predicted MW of 51.3 kDa and an isoelectric point (pI) of 5.45. The deduced protein DcCDA3 is a secreted protein because a signal peptide of 27 amino acids was found in the N-terminal region. Structural domain analysis showed that DcCDA3 has a putative chitin-binding domain (residues 39–91) and a polysaccharide deacetylase-like catalytic domain (residues 139–267). Additionally, three potential *N*-glycosylation sites (NRT, NIT, and NIS) at positions 5237, and 448 were predicted by using NetNGlyc 1.0 software (Figure 1). The phylogenetic tree analysis revealed that all insect CDAs could be divided into five groups (I–V). DcCDA3 contained chitin-binding domain (ChBD) and CDA domains, and clustered into Group ІІІ. Moreover, DcCDA3 has a close relationship with *N. lugens* CDA3 (NlCDA3) (Figure 2). In addition, five signature motifs of the deacetylase domain of DcCDA3 were identified (Figure S1). Motifs 1–5 are represented by the sequence TFDG, ETISLQ, RAPF, FIYDS, and LDHKIP.

### 2.2. Spatiotemporal Expression Patterns of DcCDA3

**Figure 3:**
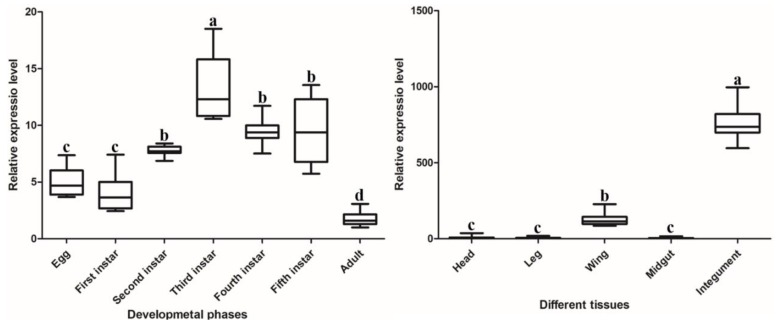
Expression patterns of *DcCDA3* at different developmental phases and in different tissues of the adult of *D. citri*. Relative mRNA levels of *DcCDA3* were analyzed using RT-qPCR. Data were normalized using glyceraldehyde-3-phosphate dehydrogenase (*GAPDH*) and are represented as the means ± standard errors of the means from three independent experiments. Relative expression levels were calculated using the 2^−∆∆*C*t^ method. Statistical analysis was conducted using SPSS 19.0 software. Significant differences are indicated by a different letter, for example, a, b, c, and d (*p* < 0.05).

The expression patterns of *DcCDA3* in different developmental stages and different tissues were investigated by RT-qPCR. The results suggested that *DcCDA3* was expressed in all *D. citri* tissues, including the head, leg, wing, midgut, and integument. It is notable that *DcCDA3* has the highest expression in the integument, followed by the wing, while it had low expression in the head, leg, and midgut. The expression level of *DcCDA3* in the integument was 123 times that in the midgut, and its expression in the wing was 20.7 times that of the midgut (Figure 3). The expression of *DcCDA3* increased from first-instar nymph to third-instar nymph, and then decreased from third-instar nymph to adult stage. However, there was no significant change from egg to the first-instar nymph stage. The expression level of *DcCDA3* in the third-instar nymph was 7.6 times that of the adult stage (Figure 3).

### 2.3. Analysis of the Expression Level of DcCDA3 after 20E Treatment

**Figure 4:**
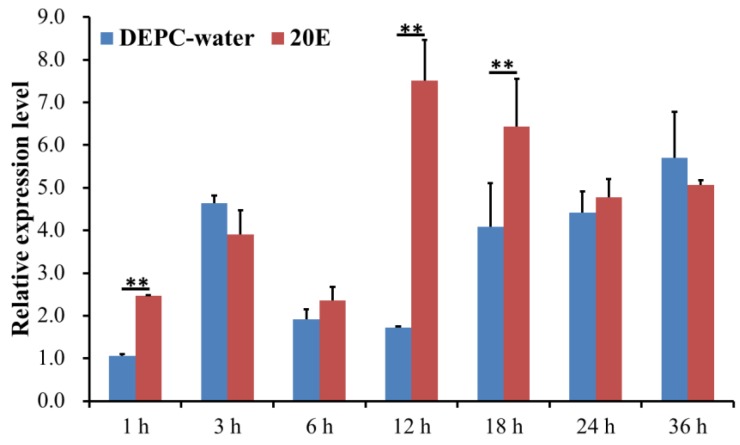
Expression levels of *DcCDA3* after 20E treatment in *D. citri*. Data were normalized using glyceraldehyde-3-phosphate dehydrogenase (*GAPDH*) and are represented as the means ± standard errors of the means from three independent experiments. Relative expression levels were calculated using the 2^−∆∆*C*t^ method. Statistical analysis was performed using SPSS software. Significant differences are indicated by (** *p* < 0.01).

To analyze whether *DcCDA3* expression can be induced by 20E treatment, the first day of fifth-instar nymphs treated with 20E were subjected to RT-qPCR. The results showed that the relative level of *DcCDA3* had an obvious fluctuation from 1 h to 36 h after 20E treatment (Figure 4). However, the expression level of *DcCDA3* was relatively low after DEPC water treatment. Compared with the control group, the larvae treated with 20E exhibited considerably increased *DcCDA3* expression levels at 12 h. These results indicated that the expression level of *DcCDA3* was affected by 20E. 

### 2.4. Functional Analysis of DcCDA3 Based on RNAi

**Figure 5:**
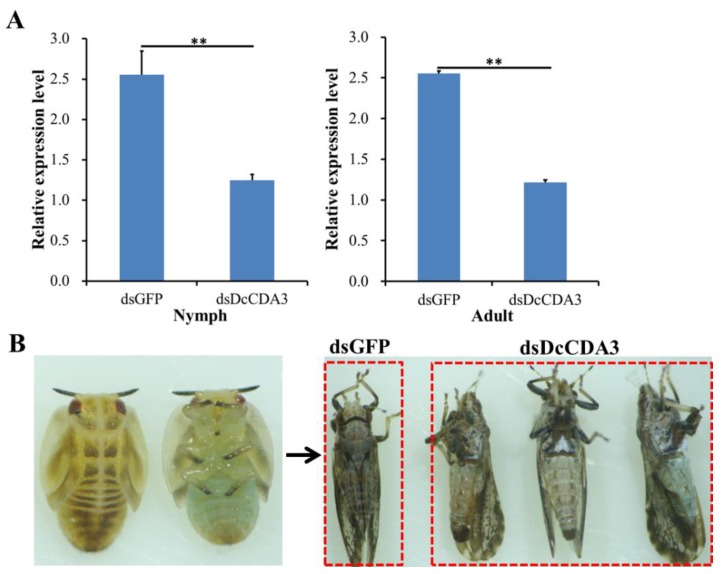
Effects on *D. citri* after RNA interference of *DcCDA3*. (**A**) Relative expression levels of *DcCDA3* when *D. citri* was treated with double-stranded *CDA3* (ds*DcCDA3*) and double-stranded green fluorescent protein (ds*GFP*). The mean expression level is represented for three biological replicates. Relative expression levels were calculated using the 2^−∆∆*C*t^ method. Statistical analysis was performed using SPSS software. Significant differences are indicated by (** *p* < 0.01). (**B**) Representative phenotypes of *D. citri* at 24 h after RNAi treatment. The left figure indicates the fifth-instar nymph, and the right figure indicates the adult after molting.

To investigate the effect of *DcCDA3* on *D. citri* molting, specific dsRNA for DcCDA3 and GFP was synthesized in vitro, and RNAi was conducted by using an artificial diet. At 24 h after feeding *D. citri* with double-stranded *DcCDA3* (ds*DcCDA3*), the relative expression level of *DcCDA3* was significantly downregulated compared with the control (dsGFP) (Figure 5A). However, all nymphs can molt normally after silencing *DcCDA3*. The adult presented a normal wing phenotype after molting (Figure 5B). 

### 2.5. Expression Profiles of DcCDA3 after Challenge with Bacteria

**Figure 6:**
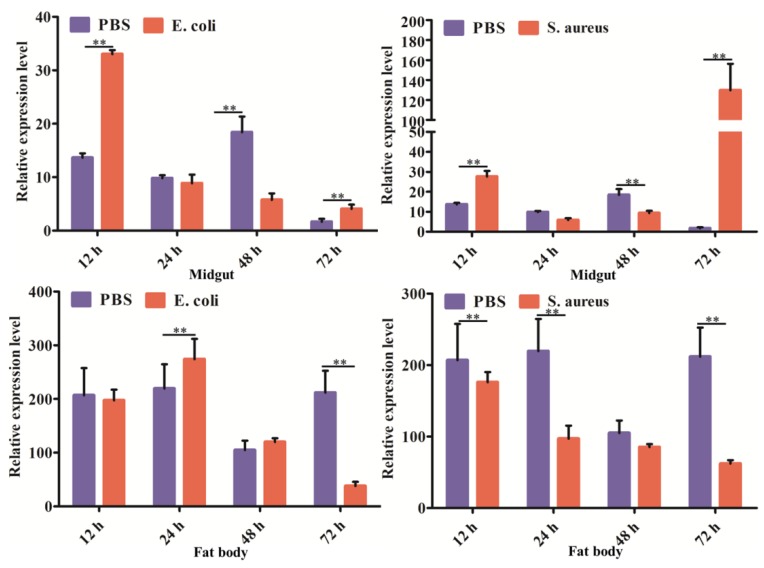
The expression levels of *DcCDA3* in the midgut and fat body after injection with *E. coli* and *S. aureus* at 12, 24, 48, and 72 h. Sterile PBS was used as a control. Relative expression levels were calculated using the 2^−∆∆*C*t^ method. Statistical analysis was performed using SPSS software. The significant differences are indicated by (** *p* < 0.01).

The expression profiles of *DcCDA3* were investigated in the midgut and fat body in response to bacterial infection using RT-qPCR at different times post-infection. In the midgut, *DcCDA3* expression was induced at the early stage of *E. coli* infection (12 h), and then decreased from 24 to 72 h after *E. coli* infection. After *S. aureus* infection, the relative expression levels of *DcCDA3* were upregulated at 12 h, and then decreased from 12 to 48 h. However, the *DcCDA3* expression level was significantly increased at 72 h post-*S. aureus* infection (Figure 6). In contrast, the relative expression level of *DcCDA3* was inhibited after bacterial infection in the fat body. At 12 to 48 h after *E. coli* challenge, the relative expression level of *DcCDA3* had no significant change in the fat body, while it was downregulated at 72 h. After a challenge with *S. aureus*, the *DcCDA3* expression level was significantly inhibited at different times in the fat body except for at 48 h. These results indicated that *DcCDA3* might be involved in the *D. citri* immune response against bacterial infection. 

### 2.6. Recombinant Protein Expression and Purification

**Figure 7:**
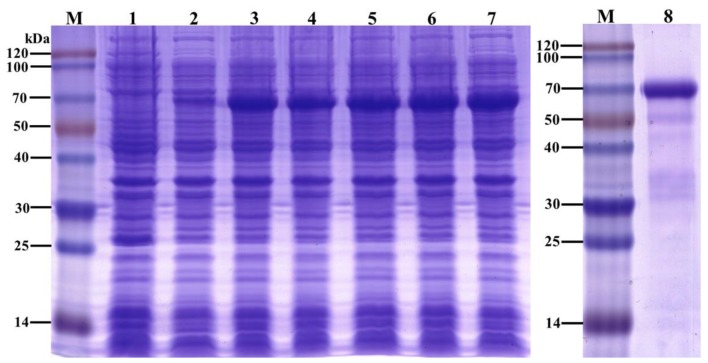
Analysis of the recombinant DcCDA3 protein using SDS-PAGE. M: molecular weight markers. Lane 1: blank control without insert. Lane 2: negative control without induction. Lanes 3–7: induced expression under Isopropyl β-d-thiogalactoside (IPTG) concentrations of 0.2, 0.4, 0.6, 0.8 and 1.0 mM, respectively. Lane 8: purified recombinant DcCDA3 protein.

To analyze the function of the DcCDA3 protein, recombinant His-tagged DcCDA3 was expressed using a prokaryotic expression system. The recombinant DcCDA3 protein with a molecular weight of approximately 70 kDa was detected by SDS-PAGE (Figure 7). The results indicated that the recombinant DcCDA3 protein was successfully expressed in *E. coli* cells. The recombinant proteins were purified and used for the next study. 

### 2.7. Enzymatic Assay forRecombinant DcCDA3 Activities

**Figure 8:**
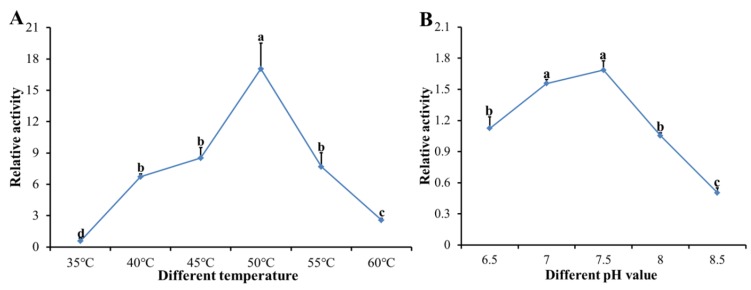
Influence of temperature and pH on the enzymatic activity of DcCDA3. (**A**) Optimal temperature and stability. (**B**) Optimal pH and stability. Significant differences are indicated by a different letter, for example, a, b, c, and d (*p* < 0.05).

The enzymatic activity of the recombinant DcCDA3 protein was assessed using 4-nitroacetanilide as the substrate and quantifying the hydrolyzed 4-nitroaniline. The results showed that the relative enzymatic activity of recombinant DcCDA3 increased from 35 to 50 °C, and then decreased from 50 to 60 °C (Figure 8A). Therefore, the optimal temperature for enzymatic activity of recombinant DcCDA3 is 50 °C. Then, we tested the optimal pH at 50 °C, as shown in Figure 8B, revealing a broad optimal pH of 6.5–8.5 with a maximum of 7.5.

### 2.8. Antibacterial Activity of Recombinant DcCDA3

**Figure 9:**
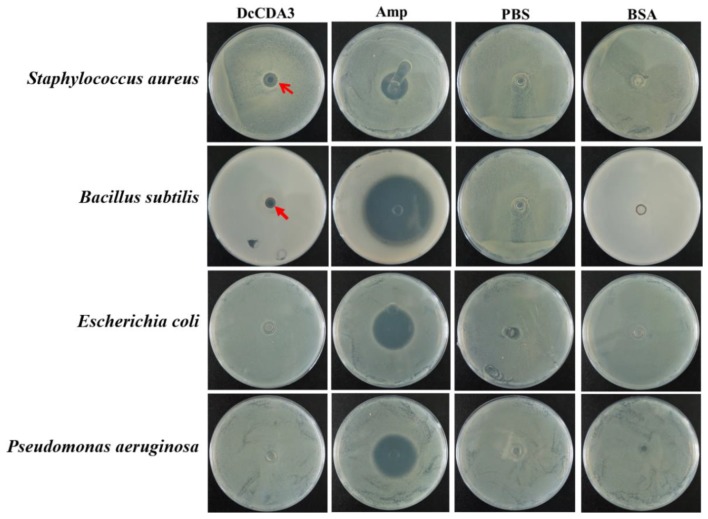
Antibacterial activity analysis of the recombinant DcCDA3 protein. No inhibition of growth was observed after treatment with bovine serum albumin (BSA) and phosphate buffer saline (PBS), which were used as negative-controls. Obvious inhibition of growth was observed after treatment with ampicillin (Amp). The red arrows indicate the obvious inhibition zone.

The antibacterial activity of recombinant DcCDA3 was assessed against *E. coli*, *P. aeruginosa*, *S. aureus*, and *B. subtilis*. The results showed that the recombinant DcCDA3 protein exhibited obvious antibacterial activity against the gram-positive bacteria *S. aureus* and *B. subtilis*, while it had no significant effect on the gram-negative bacteria. In the control group, PBS and bovine serum albumin (BSA) had no influence on the growth of bacteria. In addition, the antibacterial effect for *S. aureus* was higher than for *B. subtilis* (Figure 9). Our results suggest that recombinant DcCDA3 is active against gram-positive bacteria, but it has no influence on gram-negative bacteria. 

## 3. Discussion

Chitin is an *N*-acetyl glucosamine polymer that is involved in the formation of the protective exoskeleton of insects and is replaced periodically during insect growth and development [19]. The synthesis and degradation of chitin is essential for molting insects. During this process, three kinds of enzymes were emphasized, including chitin synthase (CHS), chitinase (CHT), and chitin deacetylase (CDA). CDAs are enzymes that catalyze the conversion of chitin into chitosan, thereby influencing the mechanism and permeability properties of structures such as the cuticle and peritrophic matrices [20]. To date, CDAs have been identified, and differences in the number of CDAs from different insect species have been shown. In *Manduca sexta*, a total of eleven CDAs were identified, with at least one representative from each of the five phylogenetic groups that have been described for CDAs to date [21]. Arakane et al. identified nine genes encoding CDAs from *Tribolium castaneum*, and RT-qPCR analysis suggested that *TcCDA1-2* and *TcCDA5* were expressed throughout all stages of development, while *TcCDA6-9* were expressed predominantly during larval feeding stages [22]. In *N. lugens*, a total of four CDAs were found based on the transcriptome and genome database [17]. In this study, we identified a *CDA3* gene from the genome database of *D. citri*. Bioinformatics analysis showed that DcCDA3 contained two conserved domains: chitin-binding domain (ChBD) and a polysaccharide deacetylase-like catalytic domain (CDA). There are two structural domains, indicating that DcCDA3 might bind to chitin and catalyze the conservation of chitin into chitosan. According to the differences in the domains, insect CDAs have been divided into five groups (I–V). Group ІІІ and Group IV contained both ChBD and CDA. In *N. lugens*, NlCDA3 also has two domains: ChBD and CDA. In recent years, CDA genes have been systematically studied, while the studies of Group ІІІ CDAs are defective. In *T. castaneum*, TcCDA4, the only Group ІІІ *CDA* gene, can be detected in the epidermal cells of imaginal appendages. The possible functions of Group ІІІ CDAs might be involved in defense against microbial pathogens. Based on phylogenetic tree analysis, DcCDA3 belongs to Group ІІІ, although the functions of DcCDA3 are unclear. 

Tissue expression patterns analysis revealed that *DcCDA3* is highly expressed in the integument, followed by the wing. The insect cuticle is most simply viewed as consisting of a thin outer epicuticle that does not contain chitin, overlying a more or less massive cuticle proper composed of chitin and protein [23]. The insect cuticle is composed mainly of chitin and chitin-binding cuticle proteins [24]. We speculated that *DcCDA3* might be involved in the degradation of chitin in the cuticle. In *N. lugens*, *NlCDA1*, *NlCDA2*, and *NlCDA4* were highly expressed in the integument [17]. In *Cnaphalocrocis medinalis*, *CmCDA5* had significantly higher expression in the integument compared with other tissues [16]. The relative expression level of *DcCDA3* increased from the egg to third-instar nymph stage and then decreased from third-instar nymph to adult stage. Additionally, we also found that the *DcCDA3* expression level could be induced by 20E. In recent years, many studies have verified that 20E can regulate the expression of chitin degradation-related genes. In *Bombyx mori*, the expression levels of *BmCDA* (1–6) were significantly increased 24 h after treatment with 20E compared with the control group [25]. Yang et al. also revealed that both *HvCDA1* and *HvCDA2* were regulated by 20E in *Heortia vitessoides* [26]. Therefore, 20E and its receptor complex ecdysone receptor and ultraspiracle (USP) play an important role in controlling insect development, metamorphosis, reproduction, and diapause. The 20E can bind ecdysteroid receptor (EcR) and USP to form a receptor complex, and then activate a small set of early-response genes. In the early stage response to 20E (for 3 to 6 h), the expression level of *DcCDA3* did not show a significant change between 20E treatment and control group, while *DcCDA3* expression level was sharply upregulated at 12 h after treatment with 20E. Taken together, our results suggest a close relationship between ecdysone and chitin metabolism. Next, RNAi was performed to verify the functions of *DcCDA3* during *D. citri* molting. After silencing *DcCDA3*, the relative expression level was significantly downregulated compared with the control group (dsGFP) in nymphs and adults. However, all nymphs can molt and do not result in abnormal molting or other interstructural abnormalities. This result seems to contradict our expectations. In *T. castaneum*, after injection of dsRNA of *TcCDA3*, no visible phenotypic changes were observed [22]. In *N. lugens*, no observable morphological or internal structural abnormalities were obtained after treatment with dsRNA for gut-specific *NlCDA3* [17]. Overall, combined with our results with related references, we considered that *DcCDA3* does not directly regulate *D. citri* molting. Chitin-containing organisms, such as fungi and arthropods, use chitin as a structural component to protect themselves from harsh environmental conditions. The expression level of *DcCDA3* was upregulated in the early stage of *E. coli* infection, while it showed high expression in the late stage of *S. aureus* infection. Annotation of the *D. citri* genome revealed that a reduced innate immune system lacking the IMD pathway is associated with defense against gram-negative bacteria, while the Toll pathway is associated with defense against gram-positive bacteria [27]. Therefore, we speculated that *DcCDA3* might be involved in defense against *E. coli* in the early stage of infection. 

To further analyze the functions of *DcCDA3*, we expressed the recombinant DcCDA3 protein using a prokaryotic expression system. In this study, the enzymatic activity of recombinant DcCDA3 protein was confirmed. The optimal temperature and pH were 50 °C and 7.5, respectively. Interestingly, recombinant DcCDA3 had a more significant effect on *B. subtilis* than *S. aureus*, while it had no obvious influence on gram-negative bacteria. In previous research, CDAs played an important role in biological attack and defense systems, and they could be used in the biological control of fungal plant pathogens or insect pests in agriculture [28]. In *Exopalaemon carinicauda*, *EcCDA1* may play its biological activity in immune defense by deacetylation from chitin [29]. Although chitin is insoluble, the partial solubility of chitosan can be produced from chitin by enzymatic deacetylation. Chitosan has been shown to possess immunostimulatory properties in mammals and plants, where it can stimulate nonspecific resistance to *E. coli* infection, suppress tumor growth, and enhance specific immunity [30]. Therefore, we hypothesized that *DcCDA3* might play an important role in the immune response of *D. citri*. 

## 4. Materials and Methods

### 4.1. Diaphorina Citri Rearing and Collection

The Asian citrus psyllid, *D. citri* were reared in a greenhouse at the National Navel Orange Engineering Research Centre, Gannan Normal University, Ganzhou, China. *D. citri* rearing was performed using uninfected *Murraya paniculata* in insect rearing cages under a 14:10 h (light: dark) photoperiod at 27–28 °C and 60–65% relative humidity. *D. citri* in different developmental stages were collected using an aspirator, including first-, second-, third-, fourth-, and fifth-instar nymphs and adults. The eggs were collected using a brush pen. A total of one hundred *D. citri* adults were dissected to obtain the midgut, head, wing, leg, and integument. All collected tissues were cleaned with pre-cooled DEPC water to remove other contents and stored at −80 °C until use. Three biological replicates of each sample were performed.

### 4.2. Bacterial Challenge and Sample Preparation

Bacterial preparation and treatment were performed as previously described [31]. Briefly, the overnight cultured *E. coli* (gram-negative) and *S. aureus* (gram-positive) cells were harvested and centrifuged at 7500× *g* for 15 min. The collected bacteria were washed three times and diluted to the desired concentrations with sterilized PBS using a hemocytometer. Bacteria were heat-killed by first diluting the cultures to the working concentrations and then placing them in a dry heat block at 95 °C for 15 min, shaking by hand every 5 min.

*D. citri* adult bacterial infection was conducted by abdominal injection. In brief, the *D. citri* adult was placed on the ice for 5 min to prevent escape, and then 30 nL (1.5 × 10^6^) of resuspended bacteria were injected into the area between thorax and abdomen. The control groups were administered with 30 nL of sterile PBS. To confirm that the bacteria were injected into *D. citri*, a brilliant blue litter was added as an indicator. Following adult infection, about fifty adults in each group were dissected at 12, 24, 48, and 72 h to collect the midgut and fat body. Briefly, *D. citri* adult abdomen was dissected using microforceps and obtained the midgut, then removed *D. citri* reproductive organs and hemolymph. Due to it is very hard to collect *D. citri* fat body by using the microforcep. Therefore, the remaining cuticle tissues containing fat body were dipped into the precooled PBS solution to elute the fat body.

The collected tissues were ground using liquid nitrogen and stored at −80 °C. Three biological replicates of each treatment were performed.

### 4.3. Preparation and Treatment of 20E

The 20E treatment of *D. citri* was performed according to the previous report with some modifications [32]. In brief, 20E (Sigma, St. Louis, MO, USA) was dissolved in 95% acetone to prepare the stock solution at a final concentration of 500 μg/μL, and then diluted to 10 μg/μL working concentrations with DEPC water. Fifth-instar nymphs were anesthetized by carbon dioxide, and then a droplet (0.2 μL) of the 20E acetone solution was applied to the prothorax of the nymph with a hand microapplicator (Sangon Biotech Co., Ltd. Shanghai, China). An acetone solution diluted with DEPC water was used as a control. The treated nymphs were collected at 1, 3, 6, 12, 18, 24, and 36 h and used for RT-qPCR analysis. Three biological replicates of each treatment were performed.

### 4.4. RNA Isolation and cDNA Synthesis

Total RNA was isolated from the adult head, midgut, integument, wing, and leg (different tissues) and first-, second-, third-, fourth-, and fifth-instar nymphs and adults (different developmental stages) by using MiniBEST Universal RNA Extraction Kit (TaKaRa, Dalian, China) according to the corresponding protocol. The integrity of the RNA was confirmed by 1.0% agarose gel electrophoresis. The RNA concentration of each sample was measured using a NanoDrop 2000 spectrophotometer (Thermo Fisher Scientific, New York, NY, USA). The purity of all RNA samples was assessed at the absorbance ratios of A_260/280_ and A_260/230_. Total RNA was reverse-transcribed in a 20 μL reaction system using First Strand cDNA Synthesis kit (Simgen, Hangzhou, China) according to a protocol. In brief, 2.0 μL of 5× gDNA Buffer and 1.0 μg of total RNA were mixed in a 100 μL PCR tube, then RNase-free H_2_O was added to reach 10.0 μL, which was then incubated at 42 °C for 3 min. Afterward, 4.0 μL of 5× RT Buffer, 2.0 μL of RT Enzyme Mix, and 4.0 μL of RT Primer Mix were added to the tube. The mixture was incubated at 42 °C for 15 min, and then incubated at 95 °C for 3 min. The cDNA was stored at −20 °C for later use.

### 4.5. Identification of DcCDA3 and Bioinformatics Analysis

DcCDA3 was identified by searching the NCBI non-redundant protein database using BLASTX (cut-off 1 × 10^−5^). Sequences of *Nilaparvata lugens CDA3* (*NlCDA3*: KJ825889.1) and *Drosophila melanogaster CDA3* (*DmCDA3*: NM_135962.3) were used as a query search of the genome database of *D. citri*. The cDNA and deduced amino acid sequence of *DcCDA3* were analyzed using DNAMAN software and BLAST (http://www.ncbi.nlm.nih.gov/blast). The signal peptide was predicted by using the SignalP4.1 Server (http://www.cbs.dtu.dk/services/SignalP). The molecular weight (MW) and isoelectric point (pI) of DcCDA3 were calculated by ExPASy (http://web.expasy.org/compute_pi). The functional domain was predicted by using SMART software (http://smart.embl-heidelberg.de/). The phylogenetic tree was constructed with MEGA 5.0 software using the neighbor-joining method with 1000-fold bootstrap resampling. Protein sequences from different insect species were downloaded from GenBank (http://www.ncbi.nlm.nih.gov/) and used for phylogenetic analysis (Table S1)

### 4.6. Reverse Transcription Quantitative PCR (RT-qPCR) Analysis of DcCDA3 Expression Levels

RT-qPCR was conducted to confirm the relative expression levels of DcCDA3 in various tissues and developmental phases. The primers were designed using Primer Premier 5.0 software (Premier Biosoft, www.premierbiosoft.com) (Table 1). The 20 μL of reaction mixture for the RT-qPCR analysis contained 10 μL of SYBR ІІ, 8 μL of ddH_2_O, 0.5 μL of forward primer, 0.5 μL of reverse primer, and 1.0 μL of cDNA template. The thermal cycling profile consisted of an initial denaturation at 95 °C for 60 s, 40 cycles at 95 °C for 10 s, 60 °C for 10 s, and 72 °C for 10 s. The reactions were performed with a LightCycle^®^96 PCR Detection System (Roche, Basel, Switzerland). Relative expression levels were calculated by using the 2^−∆∆*C*t^ method. There were three biological sample replicates, each of which included three technique replicates. The *glyceraldehyde*-*3*-*phosphate dehydrogenase* (*GAPDH*) was chosen as reference for analysis of *DcCDA3* in different tissues and different developmental phases. The samples of first-instar nymphs and head were used to normalize the data. All ANOVAS were followed by Fisher’s protected LSD tests.

### 4.7. dsRNA Synthesis and DcCDA3 RNAi Analysis

The dsRNA of DcCDA3 was synthesized using the T7 RioMAX Express RNAi System (Promega, San Luis Obispo, CA, USA) according to the manufacturer’s instructions. GFP dsRNA was used as a control. The dsRNA delivery was conducted by using an artificial diet based on the previous report. Briefly, a total of 30 fifth-instar *D. citri* nymphs were treated with ds*DcCDA3* and ds*GFP*. All nymphs were allowed to feed on an aliquot of the artificial diet (150 μL) placed between the two layers of stretched Parafilm. The artificial diet consisting of 20% (*w*:*v*) sucrose was mixed with dsDcCDA3 at a final concentration of 300 ng/μL. After 24 h, all live *D. citri* were collected to extract total RNA and synthesize cDNA. The effect of dsDcCDA3 on gene expression was investigated by RT-qPCR. A total of three biological replicates were performed for each experiment.

### 4.8. Prokaryotic Expression and Protein Purification

The specific primers (DcCDA3-Ex-F and DcCDA3-Ex-R) were designed to amply the ORF. The purified PCR product was cloned into pMD 19-T Vector (Novagen, Madison, WI, USA). Positive clones were chosen randomly for DNA sequencing to confirm the correctness of the amplified fragment. Next, the plasmid was isolated, digested, purified, and ligated into the pET-32a vector (Novagen), and then transformed into *E. coli* BL21 (DE3) (TransGen, Beijing, China) competent cells for protein expression. The protein was induced by 0.6 mM isopropyl β-d-thiogalactoside (IPTG) at 37 °C for 4 h, and the cells were harvested by centrifugation at 7500× *g* for 5 min. The cell pellets were suspended in binding buffer (20 mM Tris-HCl, 500 mM NaCl, 5 mM imidazole, pH 7.9) and disrupted by sonication on ice. After centrifugation at 12,000× *g* for 20 min at 4 °C, the recombinant proteins were purified using Ni-NTA His Bind Resin (Novagen) according to the manufacturer’s protocol. The induced and purified protein was analyzed by 12% sodium dodecyl sulfate-polyacrylamide gel electrophoresis (SDS-PAGE) and Western blotting.

### 4.9. Enzymatic Assay for Recombinant DcCDA3 Activity

The enzymatic activity of DcCDA3 was assayed using 4-nitroacetanilide as the substrate based on the previous method, and the hydrolyzed 4-nitroaniline was quantified by measuring the absorbance at 400 nm [33]. To 1 mL of the sample to be tested was added 1 mL of 200 mg/L 4-nitroacetanilide aqueous solution, and 3 mL 0.05 M (final concentration) phosphate buffer solution (pH 7.4) that was pre-incubated at 50 °C, followed by incubation in a 50 °C water bath for 30 min. The mixture was then transferred to a boiling water bath for 5 min to stop the enzymatic reaction, and distilled water was added up to 10 mL. The solution was centrifuged, and the CDA activity was determined by measuring the amount of 4-nitroaniline released from 4-nitroacetanilide at OD 400 nm. The amount of enzyme required to produce 1 μg of 4-nitroaniline per hour under the above reaction conditions was defined as one unit of CDA3 enzyme activity.

The optimum temperature of purified DcCDA3 was determined by varying the temperatures (35, 40, 45, 50, 55 and 60 °C) of the reaction mixture. The optimum pH was measured by putting reaction mixtures in different pH values (5.5, 6.0, 6.5, 7.0, and 7.5) at the optimum temperature.

### 4.10. Antibacterial Assay for Recombinant DcCDA3 Protein

The recombinant DcCDA3 protein was purified, and the recombinant DcCDA3 protein was assayed for its antibacterial activity against the gram-negative bacteria *E. coli* and *P. aeruginosa* as well as the gram-positive bacteria *S. aureus* and *B. subtilis* via the disk-diffusion susceptibility test. Briefly, the inocula (200 μL of a culture containing 1.5 × 10^6^ cfu/mL) of *E. coli*, *P. aeruginosa*, *S. aureus*, and *B. subtilis* were spread onto Luria–Bertani (LB) agar plates. The surface of the medium was allowed to dry for approximately 5 min, and then 10 μL of recombinant DcCDA3 (0.2 μg/μL) protein was added to an oxford cup. Blanks containing 10 μL of PBS or BSA (0.2 μg/μL) were used as controls. The plates were incubated at 37 °C overnight, and the resulting activities were measured by the inhibition zones. Finally, all plates were scanned using a Nikon camera (Tokyo, Japan).

## 5. Conclusions

In this study, the cDNA sequence of *DcCDA3* was identified from the genome database of *D. citri*. Gene expression analysis suggested that *DcCDA3* was highly expressed in the integument and third-instar nymph stage, and it can be induced by 20E. In response to *E. coli* and *S. aureus* injection, *DcCDA3* was upregulated in the midgut but downregulated in the fat body. After silencing *DcCDA3* by RNA interference, there was no influence on the *D. citri* phenotype. In addition, recombinant DcCDA3 possessed antibacterial activity against gram-positive bacteria. Taken together, these results lay the foundation for further research on the function of DcCDA3 and provide a new target for the control of *D. citri*. 

## Figures and Tables

**Table 1:** Primers used in this study.

Primers	Sequences	Purpose
DcCDA3-RT-F	CAGATGAGGTCTTGGAGTGGCT	RT-qPCR
DcCDA3-RT-R	GTTCTTTGCTTGACTTTGGGTTG	
GAPDH-F	CATGGCAAGTTCAACGGTGA	
GAPDH-R	CGATGCCTTCTCAATGGTGG	
ds-DcCDA3-F	TAATACGACTCACTATAGGGAGAAGGATAAGAGCAAGGAC	dsRNA synthesis
ds-DcCDA3-R	TAATACGACTCACTATAGGGTACTCGTGGGAGATGAAGAA	
ds-GFP-F	GGATCCTAATACGACTCACTATAGGCAGTGCTTCAGCCGCTACCC	
ds-GFP-R	GGATCCTAATACGACTCACTATAGGACTCCAGCAGGACCATGTGAT	
DcCDA3-Ex-F	CGGGATCCCAGAAGGATAAGAGCAA	Protein expression
DcCDA3-Ex-R	CGCTCGAGCCTGGTAGCAGAGATGC	

The underline represents the restriction enzyme cutting site.

## Supplementary Materials

The following are available online at https://www.mdpi.com/1422-0067/21/1/64/s1.

## Abbreviations

CDA	Chitin deacetylase
ACP	Asian citrus psyllid
HLB	Huanglongbing
RNAi	RNA interference
20E	20-Hydroxyecdysone
CBD	Chitin-binding peritrophin-A domain
LDLa	Low-density lipoprotein receptor class A
CHS	Chitin synthase
CHT	Chitinase
