# Supplementary material for: Immune Functional Analysis of Chitin Deacetylase 3 from the Asian Citrus Psyllid Diaphorina citri
Source: Int J Mol Sci. 2019 Dec 20;21(1):64. doi: 10.3390/ijms21010064 (PMC6981819; doi:10.3390/ijms21010064)
Supplement: Supplementary file 1 [file ijms-21-00064-s001.pdf]

Supplementary Materials

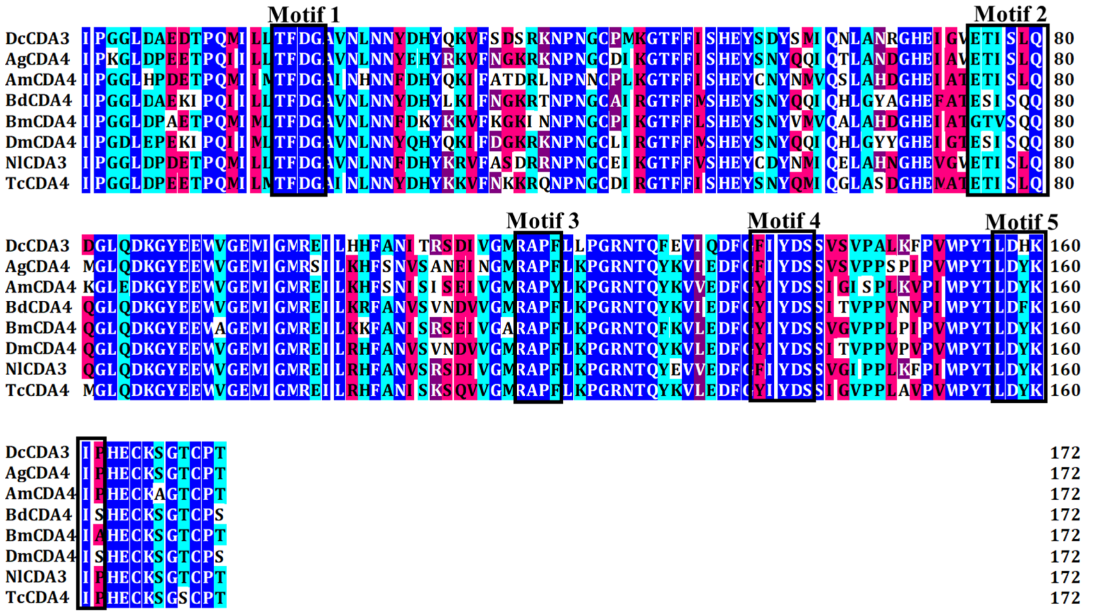

Figure 1. Amino acid sequence alignment of catalytic domains of carbohydrate esterase family 4 enzymes from eight insect species. The amino acid sequences from *Drosophila melanogaster* (Dm), *Bactrocena dorsalis* (Bd), Apis mellifera (Am), *Diaphorina citri* (Dc), *Nilaparvata lugens* (Nl), *Bombyx mori* (Bm), *Anopheles gambiae* (Ag) and *Tribolium castaneum* (Tc). The black boxes regions are the motifs (motifs 1-5) required for catalytic activity. Identical amino acids are highlighted in blue, similar amino acids are highlighted in light blue and red.

Table S1. Sequences and relevant information used for phylogenetic analysis of the chitin deacetylase gene.

**Genes**	**GenBank No**	***Species***
TcCDA2A	NP_001096047.1	*Tribolium castaneum*
TcCDA2B	NP_001116303.1	*Tribolium castaneum*
AmCDA2	XP_623723.2	*Apis mellifera*
AgCDA2	XP_320596.4	*Anopheles gambiae*
DmCDA2	NP_001163469.1	*Drosophila melanogaster*
BdCDA2A	XP_011201905.1	*Bactrocena dorsalis*
NlCDA1	AJQ20732.1	*Nilaparvata lugens*
BmCDA2	NP_001103795.1	*Bombyx mori*
BmCDA1	XP_004929283.1	*Bombyx mori*
AgCDA1	XP_320597.3	*Anopheles gambiae*
BdCDA1	AUP42574.1	*Bactrocera dorsalis*
DmCDA1	NP_001262062.1	*Drosophila melanogaster*
NlCDA2	AJQ20733.1	*Nilaparvata lugens*
AmCDA1	XP-391915.1	*Apis mellifera*
TcCDA1	NP_001095946.1	*Tribolium castaneum*
TcCDA3	NP_001104011.1	*Tribolium castaneum*
BmCDA3	XP_004931841.2	*Bombyx mori*

BdCDA3	XP_019844396.1	*Bactrocera dorsalis*
AgCDA3	XP_317336.3	*Anopheles gambiae*
DmCDA3	NP_609806.1	*Drosophila melanogaster*
DmCDA4	NP_728468.1	*Drosophila melanogaster*
BdCDA4	AUP42578.1	*Bactrocera dorsalis*
DcCDA3	XP_008480111.1	*Diaphorina citri*
NlCDA3	AJQ20734.1	*Nilaparvata lugens*
AmCDA4	XP_001120478.2	*Apis mellifera*
BmCDA4	XP_012548585.1	*Bombyx mori*
AgCDA4	XP_310753.4	*Anopheles gambiae*
TcCDA4	NP_001103903.1	*Tribolium castaneum*
BdCDA5	XP_011210019.1	*Bactrocera dorsalis*
AmCDA5	XP_624655.3	*Apis mellifera*
NlCDA4	AJQ20735.1	*Nilaparvata lugens*
TcCDA5A	NP_001103739.1	*Tribolium castaneum*
TcCDA5B	EFA13323.2	*Tribolium castaneum*
BmCDA5	XP_021207767.1	*Bombyx mori*
AgCDA5	XP_316929.4	*Anopheles gambiae*
DmCDA5	NP_001097044.2	*Drosophila melanogaster*
TcCDA7	NP_001104012.1	*Tribolium castaneum*
TcCDA8	NP_001103906.1	*Tribolium castaneum*
BmCDA9_1	XP_004923455.1	*Bombyx mori*
BmCDA9_2	XP_004923480.1	*Bombyx mori*
TcCDA9	NP_001103904.1	*Tribolium castaneum*
DmCDA9	NP_001286519.1	*Drosophila melanogaster*
BdCDA9	AUP42579.1	*Bactrocera dorsalis*
